# Not All Glittering Bone Lesions Are Gold: A Case of Sclerotic Bone Lesions with Elevated ^68^ Ga PSMA and ^99m^ Tc HDP Uptake with No Signs of Malignancy

**DOI:** 10.1055/s-0042-1758805

**Published:** 2022-12-20

**Authors:** Morten Bentestuen, Maria Carlsen Elkjær, Helle D. Zacho

**Affiliations:** 1 Department of Nuclear Medicine, Aalborg University Hospital, Aalborg, Denmark; 2 Department of Urology, Aalborg University Hospital, Aalborg, Denmark; 3 Department of Clinical Medicine, Aalborg University Hospital, Aalborg, Denmark

**Keywords:** ^68^ Ga PSMA PET/CT, bone scan, prostate cancer, primary staging

## Abstract

Gallium-68 prostate-specific membrane antigen positron emission tomography/computed tomography ( ^68^ Ga PSMA PET/CT) outperforms CT and bone scintigraphy in terms of diagnostic accuracy for the primary staging of prostate cancer and has become widely used. However, ^68^ Ga PSMA uptake is also encountered in nonprostatic tissue. We present a 63-year-old male with newly diagnosed high-risk prostate cancer who underwent bone scintigraphy with single-photon emission computed tomography/computed tomography (SPECT/CT), which showed inhomogeneous elevated uptake in sclerotic bone lesions in the pelvis. Likewise, ^68^ Ga PSMA PET/CT revealed inhomogeneous uptake in the same areas. Subsequent biopsy revealed hyperplastic bone marrow without signs of malignancy. The patient underwent radical prostatectomy, and the prostate-specific antigen level dropped to less than 0.1 ng/mL.

## Introduction

Prostate cancer is one of the most common malignancies in the Western world and frequently metastasizes to lymph nodes or bone; particularly bone metastases are the major cause of morbidity in advanced prostate cancer. 1 For primary staging of high-risk prostate cancer, gallium-68 prostate-specific membrane antigen positron emission tomography/computed tomography ( ^68^ Ga PSMA PET/CT) has proven to be a highly accurate diagnostic tool. 2

Although ^68^ Ga PSMA—as the name implies—is relatively prostate tissue-specific, several cases have demonstrated elevated ^68^ Ga PSMA uptake in benign bone lesions.

## Case History

**Fig. 1:**
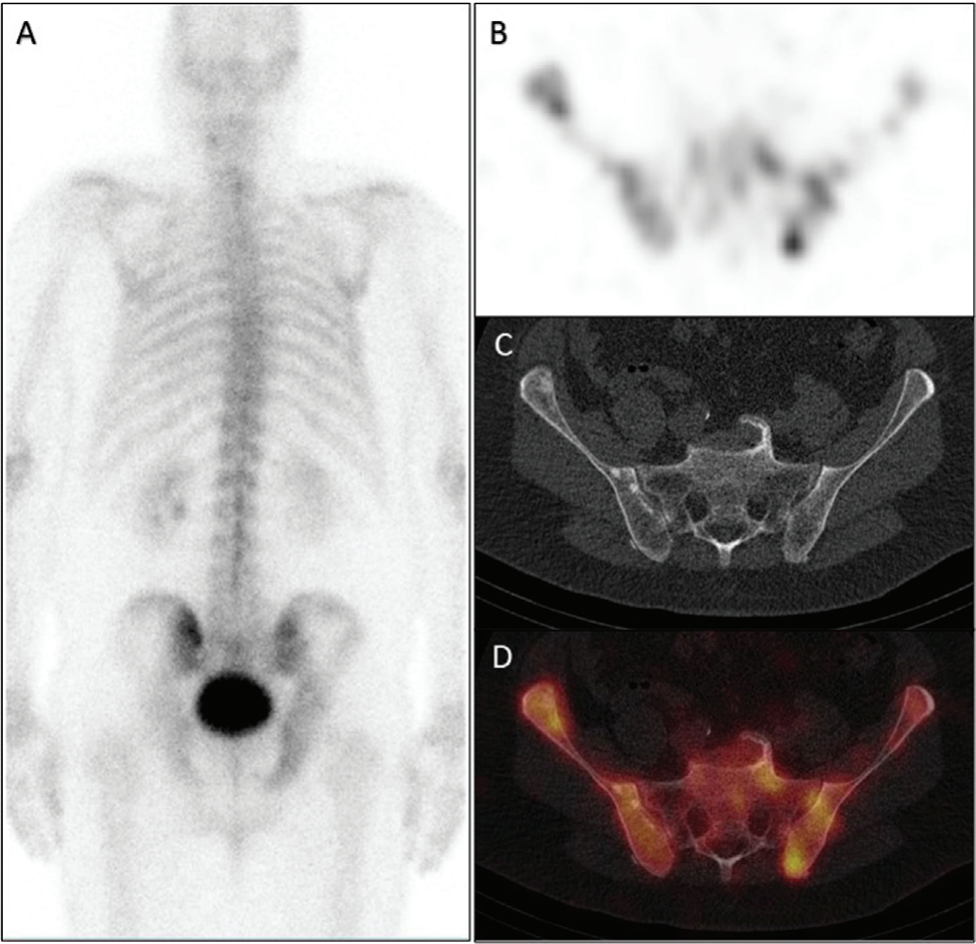
Whole body bone scan and single-photon emission computed tomography/computed tomography.

A 63-year-old male was diagnosed with high-risk prostate cancer (prostate-specific antigen [PSA] 4.7 ng/mL, Gleason score 9 [4 + 5, International Society of Urological Pathology (ISUP) grade 5], stage T1c) and referred for a ^99m^ Tc bone scan and CT of the thorax, abdomen, and pelvis. The bone scan revealed slightly elevated, inhomogeneous tracer uptake ( Fig. 1A , bone scan in posterior projection; Fig. 1B , axial single-photon emission computed tomography [SPECT]) correlating with inhomogeneous sclerotic lesions of both iliac bones on the corresponding CT ( Fig. 1C ) and as shown in the fused SPECT/CT image ( Fig. 1D ). Bone metastases were suspected, but due to the rather unusual appearance, the patient was referred to ^68^ Ga PSMA PET/CT for the further evaluation of the disease stage.

**Fig. 2:**
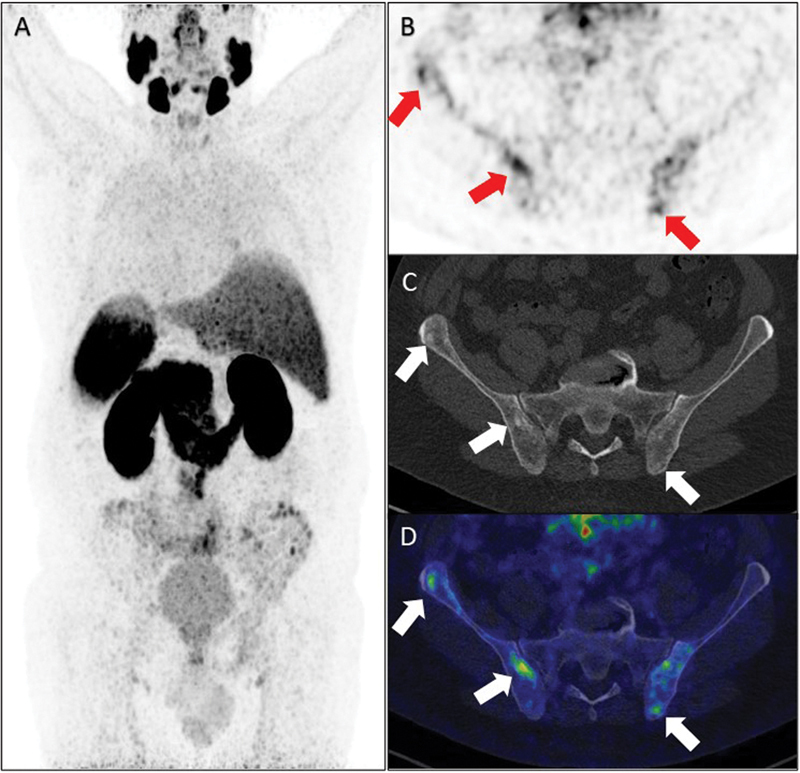
Prostate-specific membrane antigen positron emission tomography/computed tomography.

The maximal intensity projection in the posterior view of the ^68^ Ga PSMA-11 PET/CT ( Fig. 2A ) showed inhomogeneous low-to-moderate PSMA uptake (maximum standardized uptake value: 4.5–5.0) of both iliac bones, as shown in the axial PET image ( *arrows* , Fig. 2B ), corresponding to several small sclerotic lesions seen on the corresponding CT ( *arrows* , Fig. 2C ). The fused axial image is shown in Fig. 2D . In addition, the PSMA uptake pattern showed increased uptake along the iliac crest ( Fig. 2A ), which was not typical for bone metastases; for this reason, a bone biopsy was conducted to confirm or rule out bone metastases. CT-guided fine-needle and core-needle biopsies of the left ilium were conducted, and microscopic analysis revealed normal bone marrow with trilinear hyperplasia without any signs of malignancy. Immunohistochemistry analysis with the prostate-specific markers NKX3 and PSA was negative.

The patient underwent curatively intended radical prostatectomy with dissection of seminal vesicles and pelvic lymph nodes. Final pathology revealed prostate cancer pT2c N0 M0, Gleason score 7 (4 + 3), ISUP grade 3, and a spontaneous decrease in the PSA level to less than 0.1 ng/mL following prostatectomy, supporting the diagnosis of benign changes in the pelvic bones.

## Discussion

^68^ Ga PSMA PET/CT has proven to be a highly accurate diagnostic tool for staging newly diagnosed high-risk prostate cancer. 2 Despite the name prostate-specific membrane antigen, several studies have reported ^68^ Ga PSMA uptake in other types of cancer, 3 as well as several types of benign bone lesions, such as osteoid osteoma, 4 vertebral hemangioma, 5 myeloma, 6 fractures, 7 osteophytes 8 and Paget's disease. 9 Despite such findings, the specificity of ^68^ Ga PSMA PET/CT is very satisfactory and is often reported to be 93 to 98%. 2 10 However, the present case emphasizes the need for biopsy when PSMA PET/CT is not entirely diagnostic of metastases to assign the correct treatment to the patient.
